# Normalisation genes for expression analyses in the brown alga model *Ectocarpus siliculosus*

**DOI:** 10.1186/1471-2199-9-75

**Published:** 2008-08-18

**Authors:** Aude Le Bail, Simon M Dittami, Pierre-Olivier de Franco, Sylvie Rousvoal, Mark J Cock, Thierry Tonon, Bénédicte Charrier

**Affiliations:** 1 UPMC Univ Paris 6, UMR 7139 Végétaux marins et Biomolécules, Station Biologique, F 29682, Roscoff, France; 2 CNRS, UMR 7139 Végétaux marins et Biomolécules, Station Biologique, F 29682, Roscoff, France

## Abstract

**Background:**

Brown algae are plant multi-cellular organisms occupying most of the world coasts and are essential actors in the constitution of ecological niches at the shoreline. *Ectocarpus siliculosus *is an emerging model for brown algal research. Its genome has been sequenced, and several tools are being developed to perform analyses at different levels of cell organization, including transcriptomic expression analyses. Several topics, including physiological responses to osmotic stress and to exposure to contaminants and solvents are being studied in order to better understand the adaptive capacity of brown algae to pollution and environmental changes. A series of genes that can be used to normalise expression analyses is required for these studies.

**Results:**

We monitored the expression of 13 genes under 21 different culture conditions. These included genes encoding proteins and factors involved in protein translation (ribosomal protein 26S, EF1alpha, IF2A, IF4E) and protein degradation (ubiquitin, ubiquitin conjugating enzyme) or folding (cyclophilin), and proteins involved in both the structure of the cytoskeleton (tubulin alpha, actin, actin-related proteins) and its trafficking function (dynein), as well as a protein implicated in carbon metabolism (glucose 6-phosphate dehydrogenase). The stability of their expression level was assessed using the Ct range, and by applying both the geNorm and the Normfinder principles of calculation.

**Conclusion:**

Comparisons of the data obtained with the three methods of calculation indicated that EF1alpha (EF1a) was the best reference gene for normalisation. The normalisation factor should be calculated with at least two genes, alpha tubulin, ubiquitin-conjugating enzyme or actin-related proteins being good partners of EF1a. Our results exclude actin as a good normalisation gene, and, in this, are in agreement with previous studies in other organisms.

## Background

Brown algae (Phaeophyceae) are multi-cellular marine organisms that grow along temperate, tropical and polar coasts. Many of them are subject to frequent changes in their local environment, because they are uncovered at low tide, and are hence exposed to desiccation, and to variations in osmotic pressure due to rain or evaporation. In addition, pollution of the coasts, due to human activities, constitutes an additional source of abiotic stress, to which they must develop adaptive mechanisms. Expression analyses of genes involved in the perception of the stress, and in the establishment of the appropriate responses, provide a means to decipher the molecular mechanisms potentially involved in such adaptations. Despite the availability of medium scale cDNAs libraries for several different species of the brown algae (*Laminaria, Sargassum*, and *Fucus*), this task has been hindered by the lack of genome-scale resources. In 2004, Peters et al. [1] have compared a range of features in several species of Phaeophyceae and concluded that *Ectocarpus siliculosus *was the best candidate to consider for such developments. Recently, the genome of this alga has been sequenced, offering a unique opportunity to survey the expression of gene families in brown algae (Genoscope, J.M. Cock, unpublished data). *E. siliculosus *is a small filamentous alga, extensively studied over the last two centuries for its complex life cycle and its physiological features (reviewed in [2]). The genome is currently being annotated, allowing the initiation of both large scale and targeted surveys of the *Ectocarpus *genes, such as microarrays or real-time RT-PCR respectively.

Compared to high-throughput microarray techniques, real-time quantitative RT-PCR only allows assays of gene expression to be carried out at relatively low throughput (10–20 genes in 10–50 samples). Nonetheless, this technique has been adopted by a large community as a standard method for gene expression studies, because of its high reliability, and its rapidity of execution [3,4]. This technique is now widely used for a large number of animal and plant organisms, as well as for bacteria and viruses.

A few years after the emergence of this technique, a need for a reliable normalisation method became insistent. Different methods of identifying normalisation genes, such as geNorm [5], NormFinder [6] and BestKeeper [7], were then developed. This was followed by a wave of reports on the identification of the best normalisation genes from a broad range of species and specific tissues. These biological materials included human tissues [8-10] and viruses [11], as well as tissues from a variety of animals such as cow [12], pig [13], horse [14], dolphin [15], fishes [16,17], worm [18] and others. In parallel, a similar extension of the use of this technique was observed in plant research, for rice [19], poplar [20], potato [21], grapevine [22], and for plant pathogens [23].

In the brown alga *E. siliculosus*, the expression level of 20 genes specific to the two generations in the life-cycle of this alga was recently reported in a mutant impaired in development [24]. Additional developmental and physiological studies are underway, including studies aimed at assessing the resistance of this alga to environmental changes. Hence, the availability of a set of housekeeping genes for normalising the expression levels of genes of interest is a pre-requisite to any valuable conclusion, especially since this organism lives in a frequently changing environment.

In this paper, we propose optimal normalisation genes for expression analyses in *E. siliculosus*. Thirteen housekeeping genes that have been reported to be good potential candidates in the previously cited literature, were chosen for this task. Their expression was examined by Q-RT-PCR in a diversity of algal samples corresponding to growth kinetic series, osmotic stress experiments, and chemical treatments. Namely, the candidate genes are involved in the synthesis and the dynamics of the cytoskeleton, in the synthesis, folding and degradation of proteins, and in the metabolism of carbon, all of these processes being known to be only moderately affected by the fluctuation of growth conditions.

## Results

### Treatments applied to *E. siliculosus *and choice of housekeeping genes

Several different stresses were tested in this study. Chemical agents tested included H_2_O_2_, a reactive oxygen species produced by many organisms, including seaweeds, under conditions of abiotic and biotic stresses [25]. We also tested heavy metals such as copper, which are among the most significant pollution actors in marine environments worldwide. Diuron and atrazine are herbicides that inhibit photosynthesis by blocking the d1 protein of photosystem II [26,27]. Diuron is also an additive of antifouling paints, which prevent growth of organisms on ships' hulls. In addition pathogen or grazer attacks were mimicked by wounding *E. siliculosus *tissues with a razor blade. We also tested the effect of oxylipins. These are oxygenated derivatives of polyunsaturated fatty acids which play a major role in inflammatory processes, allergies, and, in a wide sense, defensive stress responses to infection, drugs, and xenobiotics [28]. In land plants, C18 derived jasmonates play a pivotal role in defense induced mechanisms [29]. In mammals, oxidation of the C20 arachidonic acid produces derivatives such as leukotriens and prostaglandins. As brown algae are able to produce oxylipins typical of both land plants and animals [30], their putative action on the induction of stress signalling pathways is of particular interest. Finally, as the metabolism and the physiology of brown algae are regulated to a large extent by diurnal rhythms, a time series of samples taken over a single day was also analysed.

RNA was extracted from biological triplicates of algae that had received the above treatments resulting in a total of 63 samples for 21 different treatments (see Table 1, and Methods for details on concentration and timing). Single strand cDNA was synthesised simultaneously from each of the 63 extracts in order to minimize any variation during this step of the process. The abundance of the transcripts of 13 potential housekeeping genes was then assayed on these cDNAs. The genes tested included commonly used genes such as a ribosomal protein and translation initiation or elongation factors (eIF2A, eIF4E, EF1a, 26S ribosomal protein), cytoskeleton proteins (tubulin alpha, actin and actin-related proteins), and proteins involved in the protein degradation process (ubiquitin and ubiquitin-conjugating enzyme). In addition, cyclophylin, two actin-related proteins, a tubulin molecular motor (dynein) and an enzyme involved in the pentose phosphate pathway (glucose 6-phosphate dehydrogenase) were included in this study. The genes tested are listed in Table 2.

**Table 1:** Culture conditions and duration.

Type of treatment	Final concentration	Duration
Diurnal cycle		0 h
Diurnal cycle		6 h
Diurnal cycle		12 h
Diurnal cycle		18 h
Diurnal cycle		24 h

ASW	450 mM NaCl	3 h
DMSO	1% (V:V)	3 h
H_2_O_2_	10 mM	3 h
CuSO_4_	10 μM	3 h
Atrazine	55 μg. L^-1^	3 h
13-HOtrE	5 μM	3 h
15-HEPE	5 μM	3 h
ASW	450 mM NaCl	6 h
Ethanol	0.2% (V:V)	6 h
CuSO_4_	10 μM	6 h
Diuron	42 μg.L^-1^	6 h
Wounding		6 h

ASW	450 mM NaCl	6 h
Hyposaline	60 mM	6 h
Hypersaline	1,5 M	6 h
H_2_O_2_	1 mM	6 h

Treatments were applied under light (see Material and Methods for details). ASW: artificial sea water; DMSO: dimethyl sulfoxide; 13-HOTrE: 13-hydroxyoctadecatrienoic acid; 15(S)-HEPE: 15-hydroxyeicosapentaenoic acid.

**Table 2:** Candidate housekeeping genes tested in this study.

**Gene symbol**	**Homologous to**	**Description of trace archive**	**Accession number**	**Oligonucleotides – Forward – Reverse**	**E (%)**	**R2**	**Tm product**	**PCR product length (bp)**
ACT	Actin	KY0AIB94YO18AHM1	1927036313	CCCAGATCATGTTCGAGACGTT CACGCCGTCACCCGAGTC	91	1.000	87.80	119
ARP2.1	p34-arc subunit of the actin-related protein complex ARP2/3	KY0AFIPA38YJ23RM1	1927195696	GAAGGAGTTCTGCCGGGAAG ACAAAGCAGCAACGCAGAGA	98	0.994	84.50	121
ARP2.2	ARP2 subunit of the actin-related protein complex ARP2/3	KY0AIB269YJ02AHM1	1929831232	GAAGAAGTTCAAGCTCAACATCGA CCGCACCCCCAATGAAA	104	0.998	80.90	68
CYC	Cyclophilin	KY0ADB29YF06FM1	1291599781	AGACGGCGGTGCAAGTAGG GTGAGTCACGGCTGCTTTTATG	92	0.997	84.80	101
Dyn	Dynein light chain protein	KY0AEB344YP09RM1	1306215256	GGAACAAAGCATGGTGACAACA CGCGTGCCTATCCAAGCT	100	0.999	81.20	65
EF1alpha	Translation elongation factor 1 alpha	KY0AEC342YI10RM1	1291335619	GCAAGGGCCTCAGCTCTG ACAAGCCGTCTGGGTATATGTTAGC	92	0.997	81.50	160
G6PD	Glucose 6 phosphate dehydrogenase	KY0AEF243YN02RM1	1299896231	GTGAGGATGTTCAGGTCCCAG GTGGAAGACCCGGTGAGGT	90	0.996	84.50	101
IF2A	Translation initiation factor eIF2 alpha	KY0AIB251YB11AHM1	1918199315	GCGGTACGTGATGGACACC CCCCCGACTCGATGATCTTT	94	0.991	84.80	101
IF4E	Translation intiation factor eIF4E	KY0ADA42YE14FM1	1291478318	TCGCGATTCGAGGTTTGAGTA CAAACGCTGCGGCAGC	100	0.991	82.40	71
R26S	Ribosomal protein 26S	KY0AEC624YL15RM1	1300144654	GCTAGGCTTGCGTTTGTGTG GGCGAGACAGAAAGATTCCG	93	0.995	85.40	101
TUA	Alpha tubulin	KY0AEC614YE14RM1	1299935912	TTTGAGGAGTTTCGTCGGAGAT CACACAGCGCAAAACGGC	92	0.999	83	140
UBCE	Ubiquitin conjugating enzyme	KY0AFIPA87YJ24RM1	1917772478	AACAATGGCCTTTGCGAAAA GCGTACGTCTTGAAGCCCAG	95	0.997	84.50	101
UBQ	Ubiquitin C	KY0AEC576YH18FM1	1306241438	CAACGCCCATGATTGTTCAC GATTATTCCCATCCACGGCA	100	0.997	82.70	101
mN	Intron amplification	KY0AEF302YN21FM1	1306150449	TCATTTTTCATGTGGAGGTCTCTG GCCAAACAAACAACAACCCTC	83	0.981	84.80	93

The identity of the sequences (gene name, function) is indicated in the left part of the table. The trace corresponds to trace archive at NCBI . Parameters on the corresponding amplified product (oligonucleotides, amplicon Tm and size, reaction efficiency and reliability) are indicated in the right part.

### Quantification and data analysis

**Figure 1:**
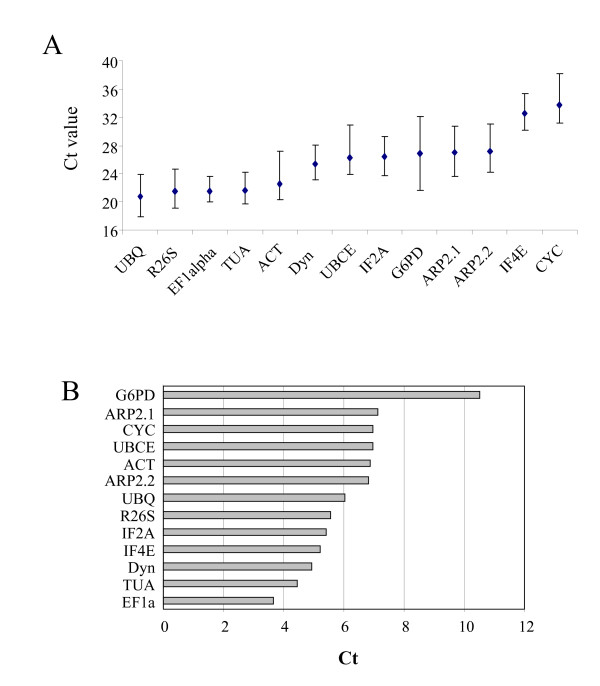
**Expression level of 13 housekeeping genes**. A: The range of the expression level of the 13 genes over the 21 culture conditions is expressed in Ct values. The black diamond represents the arithmetic mean. B: Variations observed in the range of Ct values.

In order to assess whether the transcripts of these 13 genes remained at comparable levels in the different samples tested, we calculated the variation in the Ct value for each gene. Figure 1A shows that the transcripts of these genes exhibited different levels of abundance, with *CYC *being expressed at the lowest level, and *UBQ *being expressed the most strongly. Variation in transcript accumulation across the 21 culture conditions was not the same for all the genes tested. *EF1a *showed the weakest variation, while *G6PD *expression seemed to be strongly influenced by the treatments, its range of expression level exceeding 10 Ct (Figure 1B).

**Figure 2:**
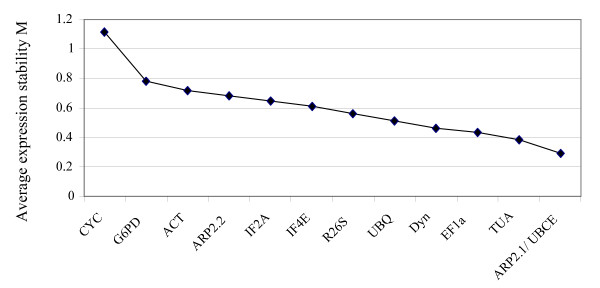
**Global ranking of the 13 housekeeping genes using geNorm analysis**. The M value was calculated with the geNorm software [5]. Low values of M indicate that a gene is expressed very stably.

In order to test the robustness of these data, we performed the geNorm pairwise analysis, which was first described by Vandesompele et al. [5], and has since been widely used to evaluate the stability of expression of genes from many organisms. The results of this analysis (Figure 2) were slightly different from those obtained with the Ct value calculation. The two calculation methods identified the same least and most stably expressed genes (*G6PD *and *CYC *were the least stable, and *TUA, EF1a *and *Dyn *were the most stable), but the intermediate genes were ordered differently. This was particularly striking for *ARP2.1 *and *UBCE*, which the geNorm analysis indicated were the most stably expressed genes, and which the Ct value analysis indicated to be among the least stably expressed. In order to test whether averaging the biological triplicates had an effect on the final result, we performed the geNorm analysis using the transcript abundances measured in the 63 individual cDNA samples, as previously described [31]. This analysis identified the same genes as having either highly variable (*CYC, G6PD *and *ACT*) or very stable (*ARP2.1, UBCE, TUA*, *EF1a *and *Dyn*) transcript abundances, but the order of the six remaining genes was again different (data not shown). Thus, averaging the biological replicates modified the results of the analysis for the intermediate genes, but not for the most and the least stable genes. A one-way ANOVA test showed that for most of the genes considered individually, the variance between the different culture conditions is significantly higher than the variance between the biological replicates for a given condition (see Additional file 1). Therefore, averaging on the three biological replicates should not introduce any significant distortion.

NormFinder is another approach that has been used to assess the stability of expression of housekeeping genes [6]. When NormFinder was applied to the data obtained in this study, it indicated that the genes with the most stable levels of transcript abundance were *TUA, ARP2.1, EF1a *and *Dyn *(Table 3). These were almost the same genes as the ones identified by geNorm, with the exception that *Dyn *performed better than *UBCE*. Therefore, there was a very good correlation between the results obtained from geNorm and NormFinder, despite the fact that the methods of calculation are fundamentally different.

**Table 3:** Normfinder analysis of the expression stability of the 13 genes.

Gene name	Stability value
TUA	0.099
ARP2.1	0.182
EF1a	0.220
Dyn	0.227
UBCE	0.240
UBQ	0.401
IF4E	0.403
IF2A	0.406
ARP 2.2	0.422
R26S	0.479
ACT	0.558
G6PD	0.655
CYC	1.964

The stability of expression of the 13 genes was calculated using the Normfinder method designed by Andersen et al.[6]

**Figure 3:**
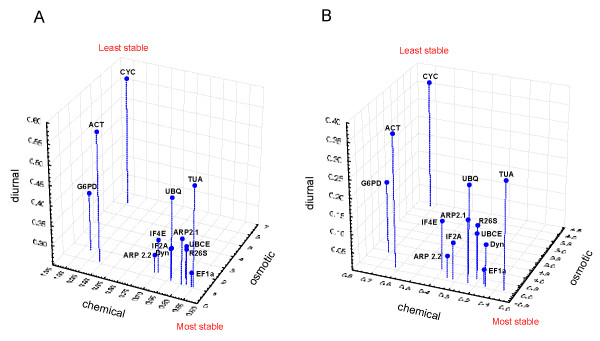
**Ranking of the 13 housekeeping genes over the three different series of culture conditions**. Both geNorm (A) and Normfinder (B) were used to order the housekeeping genes according to three axes, corresponding to the three series of culture conditions/treatments. The position of each gene in the 3-D graph indicates its suitability as a reference gene. The front bottom position corresponds to the most stable gene, the far top position to the most regulated gene. Note the different scales on the axes.

In order to test whether the normalisation genes identified above were also the best choices for specific conditions, we performed expression stability measurements on distinct series of treatments *i.e. *osmotic stresses, chemical treatments and diurnal rhythm. Figure 3 shows that with both the geNorm and NormFinder calculation methods (A and B) the optimal choice of normalisation gene depended on the type of experiments. Thus, although *EF1a *remained among the most stable genes, variations in the identity of the other very stable genes were observed depending on the treatment. This was particularly striking for the *ACT *gene, the abundance of its transcript showing a high level of variability following all the treatments except osmotic stresses. The transcript of the *TUA *gene varied significantly in abundance in the diurnal sample series (see Additional file 2), but remained at a stable level in the abiotic stress treatments. Note that *CYC *and *G6PD*, which showed the greatest variability in transcript abundance in the global analyses, were also highly variable in each of the different classes of experiment.

**Figure 4:**
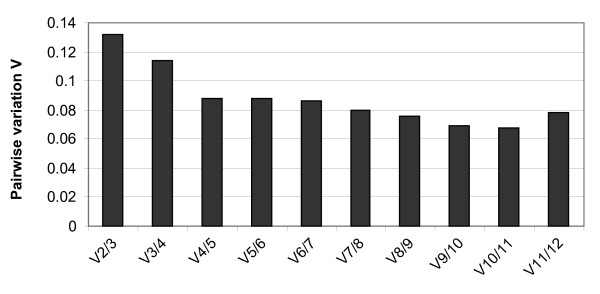
**Determination of the optimal number of control genes for normalisation**. The pairwise variation V of the normalisation factors was calculated for the 21 different culture conditions for the 13 housekeeping genes with the geNorm software [5].

To calculate the number of normalisation genes necessary to obtain the normalisation factor we determined, using geNorm, the pairwise variation between sets of normalisation factors obtained when using two, three or more genes for normalisation. Figure 4 shows that the normalisation factors are only modified slightly when a third (or more) gene is added (pairwise variation of 0.13 for 3 genes). Vandesompele et al. [5] recommended that additional normalisation genes be included if the pairwise variation between the normalisation factors is higher than 0.15. According to geNorm, measuring the expression levels of *ARP2.1 *and *UBCE *is sufficient to normalise the expression of genes of interest in these samples.

## Discussion

In this study, the three methods used to identify the best normalisation genes were concordant, as previously reported in other studies [10]. Comparison of the three methods indicates that *EF1a *is the most reliable gene to normalise gene expression in experiments aiming at quantitatively measuring the transcriptional response to abiotic stresses and chemical treatments. The V pairwise analysis shows that two genes are sufficient for a proper expression normalisation. The choice of the second gene, however, will depend on the type of experiment that is being carried out. For osmotic stresses and chemical treatments, *TUA *can be used reliably, while for the diurnal cycle *ARP2.2 *is more relevant.

As a brown alga, *E. siliculosus *is member of the kingdom of the Heterokonta, which is phylogenetically very distant from animals and land plants [32]. Interestingly, and despite this evolutionary distance, a consensus seems to emerge from similar analyses performed in organisms belonging to distant lineages. The gene coding for the elongation factor of protein translation *EF1a *was shown to be the best reference genes in salmon [17] and in several plants such as in rice [19], grapevine [22] and potato [21]. The alpha tubulin encoding gene was also reported to be one of the best reference genes for horse tissues [13] and in poplar [19]. On the other hand, actin genes have been very often reported as exhibiting highly variable levels of expression in both human and animals tissues [10,12,16-18], and in plants [19]. In this study, we have shown that this is also true for *E. siliculosus*.

Despite the fact that ubiquitin and related enzymes (UBCE) are not commonly used as normalisation genes, in this study, they were found to be quite suitable. Interestingly, Czechowski et al. [31] showed, using microarray analysis, that genes of the ubiquitin complex, comprising an ubiquitin conjugating enzyme such as UBCE, and several E3-ubiquitin protein ligases, were very stably expressed. They also pointed out that genes with fairly low levels of expression such as *UBCE *may be of particular interest for normalising expression levels of genes that are expressed at moderate to low levels, such as transcription factors. This latter example illustrates how microarray analyses may be useful to find additional normalisation genes, which can be then tested by Q-PCR for their suitability.

## Conclusion

*E. siliculosus *is recognised as the genomic and genetic model of brown macroalgae [1,2]. As the genome sequence is currently in the phase of expert annotation, the community interested in *E. siliculosus *is likely to grow in the near future. The results presented in this paper pave the way for further studies on different aspects of *E. siliculosus *biology including development/morphogenesis and abiotic/biotic stress responses. In addition, they will be helpful for comparison with results from microarray hybridizations, which are currently in progress.

## Methods

### Culture conditions and treatments

*E. siliculosus *(Ectocarpales, Phaeophyceae) unialgal strain 32 (CCAP accession 1310/4, origin san Juna de Marcona, Peru) was cultivated in 10 L plastic flasks in a culture room at 14°C using filtered and autoclaved natural seawater enriched in Provasoli nutrients [33]. Light was provided by Philips daylight fluorescence tubes with a photon flux density of 40 μmol. m-^2 ^.s^-1 ^for 14 hours per day. Cultures were bubbled with filtered (0.22 μm) compressed air to avoid CO_2 _depletion. To conduct the chemical treatment experiments, algal tissues were transferred into Petri dishes containing artificial seawater enriched with Provasoli (ASW) for at least 18 h before treatments in order to acclimatize the cultures to the change of growth conditions. They were then treated with different chemicals for 3 and/or 6 h (see Table 1) during the light phase. The treatments were 10 mM H_2_O_2 _(final concentration), 42 μg.L^-1 ^Diuron, 55 μg.L^-1 ^atrazine, 5 μM 13-HOTrE and 15(S)-HEPE. Algae were also incubated in 10 μM CuSO_4 _for 3 and 6 h. An equal volume of solvent (ASW or DMSO 1%) was used in each corresponding control treatment. Wounding was carried out by damaging the tissues with a razor blade. To perform saline stress and an additional H_2_O_2 _treatment, tissues were transferred to ASW for one week before applying the stress. The NaCl concentration in control ASW was 450 mM, while it was 60 mM and 1.5 M in ASW used to submit the alga to hyposaline and hypersaline conditions respectively. In addition, H_2_O_2 _was added at 1 mM final concentration to the control ASW for generating oxidative stress. Treatments were applied for 6 h before harvesting the tissues for RNA extraction. To collect samples through the diurnal cycle, algae were incubated in ASW and tissue harvested every 6 h during one day. The first sample was taken 30 min after the beginning of the light period. The summary of the culture conditions is presented in Table 1.

Three biological replicates were obtained for each treatment and these were used for total RNA extraction.

### RNA extraction

The protocol used for RNA extraction was based on the method developed by Apt et al. [34] with some modifications. Frozen tissue was ground in liquid nitrogen and immediately incubated in the presence of extraction buffer (100 mM Tris-HCl pH 7.5, 2% CTAB, 1.5 M NaCl, 50 mM EDTA, 50 mM DTT). The sample was shaken at room temperature for 30 min, then one volume of chlorophorm:isoamylic alcohol (24:1, V/V) was added and the sample shaken again for 25 min. After centrifugation, the upper phase was transferred to a new tube and mixed with 0.3 V of absolute ethanol to precipitate the polysaccharides, and extracted with 1 V of chlorophorm. After centrifugation the upper phase was transferred to a fresh tube and RNA was precipitated by addition of 0.25 V of 12 M LiCl and β-mercapto-ethanol to 1% final concentration, overnight at -20°C. After centrifugation, the pellet was resuspended for DNAse treatment by an RNAse-free DNAse I (Turbo DNAse, Ambion) according to the manufacturer's instructions, in order to eliminate any residual genomic DNA from the preparation. An extraction was then carried out by adding Phenol-Chlorophorm (1:1, V/V). After centrifugation the upper phase was transferred to a fresh tube, and extracted with 1 V of chlorophorm:isoamylic alcohol (24:1, V/V) and centrifuged again. The upper phase was precipitated with 0.3 M NaAc pH 5.5 and 75% ice cold ethanol by incubating overnight at 20°C. After centrifugation, the supernatant was removed, and the pellet washed with 80% ethanol. After centrifugation and drying on ice, the pellet was resuspended in an appropriate volume of RNAse-free water.

### Quantification of RNAs and cDNA synthesis

Nucleic acid concentrations were measured by the absorbance at OD_260 _using a NanoDrop ND-1000 spectrophotometer. The purity of the RNA samples was assessed by measuring the ratio OD_260_/OD_280 _and OD_230_/OD_260 _(see Additional file 3). RNA integrity was then verified on 1.5% agarose gel stained with ethidium bromide (see Additional file 4). From each RNA sample, 1.4 μg was reverse transcribed to cDNA using oligo(dT)_12–18 _and the Superscript™ First Strand synthesis for RT-PCR (Invitrogen) according to the manufacturer's instructions, and subsequently diluted with nuclease free water to 1 ng.μl^-1 ^cDNA.

### Protocol for DNA extraction

Frozen tissue was ground in liquid nitrogen and then in a wheaton potter with 15 ml of extraction buffer (100 mM Tris-HCl pH7.5; 1.5 M NaCl; 2% CTAB; 50 mM EDTA; 50 mM DTT) per g of wet tissue. The suspension was then mixed vigorously at room temperature for at least 30 min. Proteins were degraded with 25 units of proteinase K for 2 h at 55°C, and then extracted with chlorophorm/isoamyl alcohol for several minutes. Polysaccharides were precipitated with progressive addition of 0.2 – 0.3 V of ethanol, and then extracted with 1 V of chloroform after spinning at 10,000 g and 20°C for 20 min. Nucleic acids were recovered from the upper phase by addition of 0.25 V of 12 M LiCl and 1% of β-mercaptoethanol, incubation at -20°C overnight and spinning at 10,000 g and 4°C for 1 h. The supernatant was precipitated with 0.6 V isopropanol, 0.3 M NaAc pH 5.2, left at 4°C for 30 min, and then spun down for 30 min at 13,000 g. The pellet was then dissolved in 400 μl H_2_O, and precipitated again with ethanol and NaAc. DNA was dissolved in 500 μl of TE (10 mM Tris-HCl pH 8.0; 1 mM EDTA), 5.4 M CsCl (density 1.66) and 250 μg.mL^-1 ^of ethidium bromide. Spinning at 90,000 g for 24 h at room temperature allowed the recovery of a band containing ultrapure genomic DNA under UV. Ethidium bromide was extracted 4–5 times with TE-saturated butanol and CsCl eliminated by successive ethanol precipitations.

### Real-time PCR

All the genes were quantified on the same lot of cDNAs, to minimize experimental variation that could be due to cDNA synthesis. For each gene, a pair of oligonucleotide sequences was designed in the 3' UTR of the genes when the sequence was known, or in the 3'coding sequence using Primer Express TM1.0 (PE Applied Biosystems, Foster City, CA, USA) (Table 2). The Q-PCR reactions were performed in a 96-well thermocycler (Biorad, Opticon) with SYBRgreen reaction mix from ABgene (AB-1162/B), for 15 min at 95°C, followed by 41 runs of 15 sec at 95°C and 1 min at 60°C. Each sample was technically duplicated. *E. siliculosus *genomic DNA was used as a quantification reference. A dilution series ranging from 37 to 48671 copies of the *E. siliculosus *genome was prepared and tested for each gene. The amplification efficiency was tested using this dilution series (Table 2). The specificity of amplification was checked with a dissociation curve obtained by heating the samples from 65°C to 95°C (Table 2). In addition to the DNAse-I treatments of RNAs, the absence of a genomic DNA contaminants was checked, by amplifying an intron sequence on the cDNAs. The number of copies of contaminant gDNA was subtracted from all other values, prior to any further analyses.

## Abbreviations

13-HOTrE: 13-hydroxyoctadecatrienoic acid; 15(S)-HEPE: 15-hydroxyeicosapentaenoic acid; ACT: actin; ARP2: actin-related protein 2/3; ASW: artificial sea water; CYC: cyclophilin; DMSO: dimethyl sulfoxide Dyn: dynein; EF1a: translation elongation factor alpha; G6PD: glucose 6-phosphate dehydrogenase; IF2A: translation initiation factor 2 A; IF4E: translation initiation factor 4E; R26S: ribososomal protein 26S; TUA: tubulin alpha; UBCE: ubiquitin conjugating enzyme; UBQ: ubiquitin.

## Authors' contributions

P-O dF, SR and SD extracted the RNAs. SR provided *Ectocarpus *genomic DNA. TT tested the quality of the RNAs and performed the cDNA synthesis. MC gave access to the *E. siliculosus *genomic resources. BC chose the housekeeping genes, found homologues in the *Ectocarpus *databases and designed the oligonucleotides for Q-PCR. ALB and SD carried out the Q-PCR experiments. Analyses of the data were performed equally by ALB, SD, TT & BC. The MS was written by TT and BC, and approved by all the authors.

## Supplementary Material

Additional file 1 One-way ANOVA test for the significance of the biological triplicate averaging. An ANOVA was performed on the three biological replicates of all the data, with the groups corresponding to the different treatments. The resulting p-values are shown in the table. Click here for file

Additional file 2 Gene expression during the diurnal cycle. Click here for file

Additional file 3 Quantification and quality of the RNAs used in this study. Click here for file

Additional file 4 RNA extracts considered in this study. From the 83 RNAs run on the gel, the ones used for the cDNA synthesis are labelled in red. Between 400 to 900 ng of RNA were loaded on the gel. Click here for file
