# Supplementary material for: Normalisation genes for expression analyses in the brown alga model Ectocarpus siliculosus
Source: BMC Mol Biol. 2008 Aug 18;9:75. doi: 10.1186/1471-2199-9-75 (PMC2546422; doi:10.1186/1471-2199-9-75)
Supplement: Additional file 1 — One-way ANOVA test for the significance of the biological triplicate averaging. An ANOVA was performed on the three biological replicates of all the data, with the groups corresponding to the different treatments. The resulting p-values are shown in the table. [file 1471-2199-9-75-S1.pdf]

Gene	p-value
R26S	1,85E-01
ARP2.1	9,11E-04
UBCE	6,75E-04
UBQ	7,47E-05
IF4E	4,37E-05
EF1alpha	1,56E-05
Dyn	5,40E-06
TUA	1,47E-06
ACT	1,18E-07
IF2A	3,12E-07
CYC	9,53E-08
ARP2.2	1,07E-08
G6PD	1,11E-10

p-value for rejecting the hypothesis that gene expression is identical among the tested conditions, tested by a one-way ANOVA
