# Supplementary material for: Normalisation genes for expression analyses in the brown alga model Ectocarpus siliculosus
Source: BMC Mol Biol. 2008 Aug 18;9:75. doi: 10.1186/1471-2199-9-75 (PMC2546422; doi:10.1186/1471-2199-9-75)
Supplement: Additional file 3 — Quantification and quality of the RNAs used in this study. [file 1471-2199-9-75-S3.pdf]

	Sample	Nanodrop ng/ul	Nanodrop 260/280	Nanodrop 260/230	gel ul	**gel sample number**
diurnal cycle	t0 a	612	1.97	2.35	1	**1**
	t0 b	784	2.07	2.4	1	**2**
	t0 c	514	2.06	2.36	1	**3**
	t6 a	576	2.08	2.37	1	**4**
	t6 b	712	2.1	2.43	1	**5**
	t6 c	605	2.04	2.32	1	**6**
	t12 a	634	2.04	2.36	1	**7**
	t12 b	748	2.09	2.45	1	**8**
	t12 c	591	2.04	2.39	1	**9**
	t18 a	710	2.08	2.39	1	**10**
	t18 b	685	2.08	2.42	1	**11**
	t18 c	580	2.07	2.39	1	**12**
	t24 a	818	2.11	2.42	1	**13**
	t24 b	752	2.11	2.37	1	**14**
	t24 c	698	2.07	2.34	1	**15**
chemical treatment 1	SW 3h00 A	1152.2	2.13	2.31	0.75	**16**
	SW 3h00 B	554.3	2.13	2.32	1	**17**
	SW 3h00 C	500.1	2.17	2.37	1	**18**
	H2O2 3h00 A	357.5	2.21	2.52	2	**19**
	H2O2 3h00 C	1044.6	2.06	2.27	0.75	**20**
	H2O2 3h00D	457.2	2.09	2.31	1.5	**21**
	CuSO4 3h00 A	511.8	2.14	2.32	1	**22**
	CuSO4 3h00 B	439.1	2.17	2.45	1.5	**23**
	CuSO4 3h00 C	1054.8	2.12	2.33	0.75	**24**
	DMSO 3h00 A	416.9	2.19	2.53	1.5	**25**
	DMSO 3h00 B	548.2	2.13	2.34	1	**26**
	DMSO 3h00 D	487.5	2.18	2.38	1.5	**27**
chemical treatment 2	SW 6h00 A	410.6	2.08	1.96	1.5	**30**
	SW 6h00 B	399.4	2.12	2.16	1.5	**31**
	SW 6h00 C	409.6	2.12	2.05	1.5	**32**
	CuSO4 6h00 A	1243.2	2.20	2.44	0.5	**36**
	CuSO4 6h00 C	494.6	2.13	2.18	1.5	**37**
	CuSO4 6h00 D	956.9	2.08	2.21	0.75	**38**
	Diuron 6h00 A	993.8	2.14	2.34	0.75	**42**
	Diuron 6h00 B	1551.7	2.12	2.31	0.5	**43**
	Diuron 6h00 C	459.7	2.15	2.34	1.5	**44**
	wounding 6h00 A	861.2	2.14	2.34	1	**48**
	wounding 6h00 C	392.4	2.12	2.33	2	**49**
	wounding 6h00 D	1266.1	2.09	2.31	0.5	**50**
	ethanol 6h00 A	511.5	2.12	2.30	1.5	**53**
	ethanol 6h00 B	1094.7	2.13	2.34	0.5	**54**
	ethanol 6h00 D	392.8	2.12	2.25	2	**55**
	atrazine 3h00 A	1086.9	2.12	2.39	0.75	**56**
	atrazine 3h00 C	1207.1	2.10	2.32	0.75	**57**
	atrazine 3h00 D	1336.5	2.12	2.38	0.5	**58**
	13-HOtrE 3h00 B	898.0	2.12	2.34	1	**65**
	13-HOtrE 3h00 C	450.1	2.12	2.34	1.5	**66**
	13-HOtrE 3h00 D	731.4	2.0	2.27	1	**67**
	15-HEPE 3h00 B	1149.6	2.13	2.34	0.75	**69**
	15-HEPE 3h00 C	362.4	2.1	2.32	2	**70**
	15-HEPE 3h00 D	748.5	2.08	2.17	1	**71**
Abiotic stress samples 6h00	control	292.2	2.11	2.42	2.5	**75**
	control	263.5	2.12	2.44	2.5	**76**
	control	235.1	2.11	2.58	2.5	**77**
	hyposalin	261.0	2.11	2.40	2.5	**78**
	hyposalin	313.0	2.12	2.41	2.5	**79**
	hyposalin	279.7	2.11	2.55	2.5	**80**
	hypersalin	264.4	2.09	2.57	2.5	**81**
	hypersalin	223.5	2.10	2.57	2.5	**82**
	hypersalin	350.4	2.12	2.36	2.5	**83**
	H2O2	233.6	2.06	2.45	2.5	**84**
	H2O2	250.6	2.11	2.50	2.5	**85**
	H2O2	353.2	2.11	2.46	2.5	**86**
