# Supplementary figures and images for: Normalisation genes for expression analyses in the brown alga model Ectocarpus siliculosus
Source: BMC Mol Biol. 2008 Aug 18;9:75. doi: 10.1186/1471-2199-9-75 (PMC2546422; doi:10.1186/1471-2199-9-75)

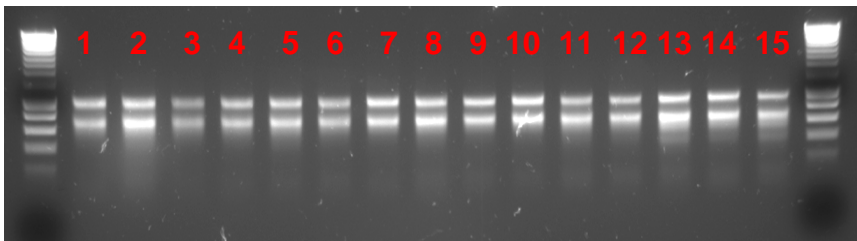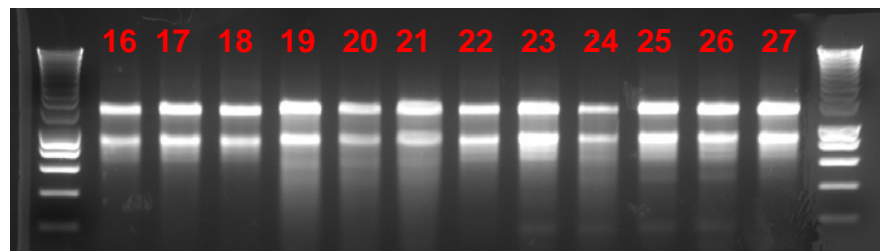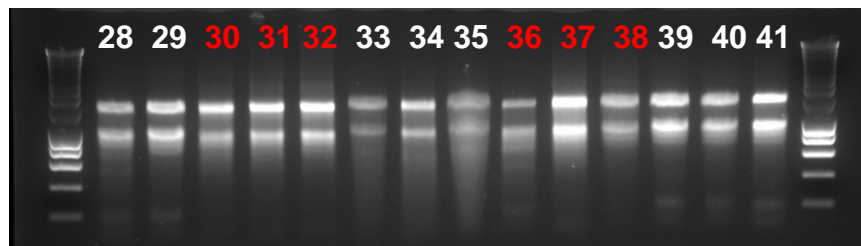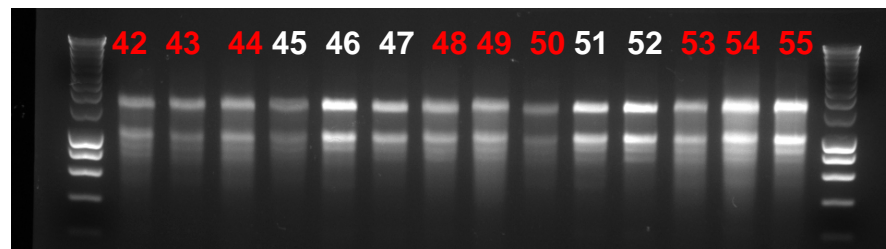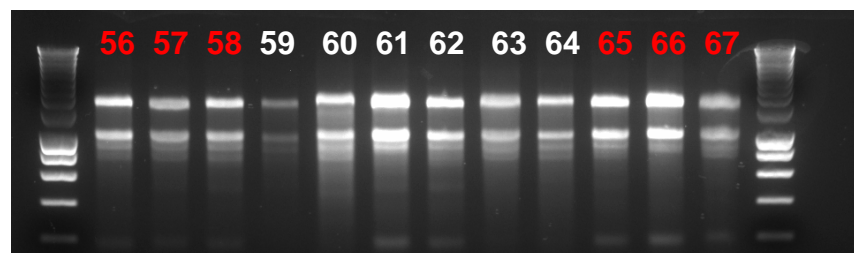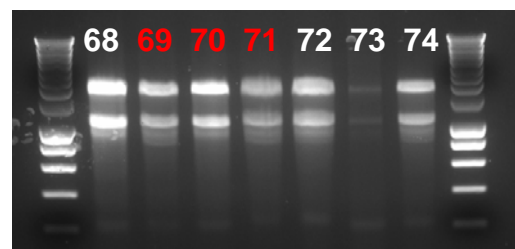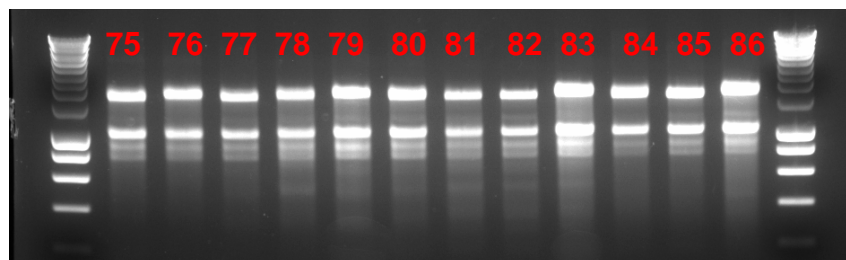

Supplement: Additional file 4 — RNA extracts considered in this study. From the 83 RNAs run on the gel, the ones used for the cDNA synthesis are labelled in red. Between 400 to 900 ng of RNA were loaded on the gel. [file 1471-2199-9-75-S4.pdf]
